# rBC2LCN lectin as a potential probe of early‐stage HER2‐positive breast carcinoma

**DOI:** 10.1002/2211-5463.12852

**Published:** 2020-05-05

**Authors:** Shuuji Mawaribuchi, Yoshikazu Haramoto, Hiroaki Tateno, Yasuko Onuma, Yasuhiko Aiki, Yuzuru Ito

**Affiliations:** ^1^ Biotechnology Research Institute for Drug Discovery National Institute of Advanced Industrial Science and Technology (AIST) Tsukuba Japan

**Keywords:** breast carcinoma, epithelial‐to‐mesenchymal transition, HER2, lectin, rBC2LCN

## Abstract

The recombinant N‐terminal domain of BC2L‐C lectin (rBC2LCN) is useful for detecting not only human pluripotent stem cells but also some cancers. However, the cancer types and stages that can be detected by rBC2LCN remain unclear. In this study, we identified the human breast carcinoma subtypes and stages that can be detected by rBC2LCN. Compared with rBC2LCN‐negative breast carcinoma cell lines, the rBC2LCN‐positive cells expressed higher levels of human epidermal growth factor receptor 2 (*HER2*) and epithelial marker genes. Importantly, rBC2LCN histochemical staining of human breast carcinoma tissues demonstrated the utility of rBC2LCN in detecting breast carcinoma types that express HER2 and have not spread much in the early phase of growth. We conclude that rBC2LCN may have potential as a detection probe and a drug delivery vehicle to identify and treat early‐stage HER2‐positive breast carcinoma.

## Graphical abstract

We examined human breast carcinoma cell lines and tissues using The recombinant N‐terminal domain of BC2L‐C lectin (rBC2LCN). The expression levels of *FUT1*, human epidermal growth factor receptor 2 (*HER2*), and epithelial marker genes were higher in rBC2LCN‐positive than in rBC2LCN‐negative breast carcinoma cell lines. rBC2LCN can be used to specifically detect early‐stage HER2‐positive breast carcinoma tissues. Therefore, rBC2LCN may have potential as a detection probe and a drug delivery carrier for early‐stage HER2‐positive breast carcinoma.

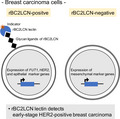

**Abbreviations**
AR: androgen receptor
B3GalT5: beta‐1,3‐galactosyltransferase 5
ER: estrogen receptor
FUT1: fucosyltransferase 1
GO: gene ontology
HER2: human epidermal growth factor receptor 2
PR: progesterone receptor
rBC2LCN: recombinant N‐terminal domain of BC2L‐C lectin from *Burkholderia cenocepacia*
TNM: tumor node metastasis

Globally, breast cancer is one of the most common cancers in women that can be treated with lumpectomy, mastectomy, radiation therapy, chemotherapy, and so on [1, 2, 3]. Defining the characteristics of breast cancer is important for classifying its subtypes, understanding the disease prognosis, and determining a suitable therapy. Estrogen receptor (ER), progesterone receptor (PR), human epidermal growth factor receptor 2 (HER2), and Ki67 are the known prognostic and predictive markers for breast cancer [1, 2, 3]. According to immunohistochemical and gene expression studies on ER, PR, HER2, and Ki67, human breast cancers are classified into the following subtypes: luminal A‐like (ER+/PR+/HER2−/low Ki67), luminal B/HER2‐negative‐like (ER+/PR+−/HER2−/high Ki67), luminal B/ HER2‐positive‐like (ER+/PR+−/HER2+), HER2‐positive (ER−/PR−/HER2+), and triple‐negative (ER−/PR−/HER2−) [1, 2, 3]. This subtype classification is essential for determining a suitable therapy for patients with breast cancer. The hormone therapies that target the ER include tamoxifen and aromatase inhibitors, which have been used to treat ER‐positive breast cancer [3]. In addition, anti‐HER2 therapies such as the monoclonal antibody drugs trastuzumab and pertuzumab and the receptor tyrosine kinase inhibitor lapatinib have been employed for the treatment of HER2‐positive breast cancer [3].

Lectins are carbohydrate‐binding proteins that are found in animals, plants, viruses, bacteria, and fungi [4, 5, 6]. Lectins are anticipated to be utilized for the diagnosis and therapy of cancer, as well as for drug delivery to cancerous cells [5, 6, 7]. The recombinant N‐terminal domain of the BC2L‐C lectin (rBC2LCN) from *Burkholderia cenocepacia* binds specifically to Fucα1‐2Galβ1‐3GlcNAc (GalNAc)‐containing glycans, such as H type 1 (Fucα1‐2Galβ1‐3GlcNAc), H type 3 (Fucα1‐2Galβ1‐3GalNAc), Lewis b (Fucα1‐2Galβ1‐3(Fucα1‐4)GlcNAc), and Globo H (Fucα1‐2Galβ1‐3GalNAcβ1‐3Galα1‐4Galβ1‐4Glc) [8, 9]. We have previously reported that rBC2LCN can discriminate the undifferentiated status of human pluripotent stem cells [9, 10]. In addition, rBC2LCN lectin has been shown to detect some human cancers, including breast, pancreatic, and prostate cancers [7, 11, 12]. Our most recent data suggested that rBC2LCN was useful for detecting a cancer stem‐like subpopulation of human prostate carcinoma PC‐3 cells [12].

Fucosyltransferase 1 (FUT1) was suggested to play important roles in the growth regulation, adhesion, migration, and cancer stem cell properties of human breast carcinoma cells *in vitro* [13]. The glycans detected by rBC2LCN are synthesized by beta‐1,3‐galactosyltransferase 5 (B3GalT5) and FUT1/2 [9]. Therefore, rBC2LCN may be useful for the detection and targeted therapy of breast cancer. In this study, we examined the human breast cancer subtypes and stages that can be detected by rBC2LCN using human breast carcinoma cell lines and tissues.

## Materials and methods

### Cell culture

Breast carcinoma cell lines MCF‐7 (HTB‐22), T‐47D (HTB‐133), MDA‐MB‐157 (HTB‐24), and MDA‐MB‐231 (HTB‐26) were obtained from ATCC (Manassas, VA, USA) and were maintained according to the providers’ instructions. The culture media and conditions of the cell lines used in this study are shown in Table S1.

### Flow cytometry

The rBC2LCN lectin was prepared, as previously described [9]. rBC2LCN and BSA (A7638‐10G; Sigma, St. Louis, MO, USA) were labeled using the HiLyte Fluor 647 Labeling Kit‐NH2 (LK15; Dojindo, Kumamoto, Japan), as described in a previous paper [10]. Flow cytometry was performed using HiLyte Fluor 647‐conjugated rBC2LCN or HiLyte Fluor 647‐conjugated BSA, as previously described [12]. The flow cytometry data were acquired on FACSAria (BD Biosciences, Franklin Lakes, NJ, USA) and SH800Z (Sony, Tokyo, Japan) devices and were analyzed using FlowJo v10 software (BD Biosciences).

### DNA microarray analysis

DNA microarray analysis was performed, as previously described [10, 12]. Raw microarray data were submitted to the Gene Expression Omnibus at the National Center for Biotechnology Information (accession number http://www.ncbi.nlm.nih.gov/geo/query/acc.cgi?acc=GSE139670). The data were analyzed using GeneSpring GX14.9 software (Agilent, Santa Clara, CA, USA) after applying two normalization procedures, including (a) setting of < 1 signal intensities to 1 and (b) normalization of each chip to the 75th percentile of all measurements from that chip. The baseline transformation of these data was not performed. Volcano plot, heat map, and clustering analyses were performed using GeneSpring GX14.9 software (Agilent). Gene ontology (GO) enrichment analysis was carried out using the PANTHER overrepresentation test (http://geneontology.org).

### Human breast carcinoma tissue microarray

Human breast carcinoma tissue microarray was purchased from Cybrdi (CC08‐10‐001; Cybrdi, Gaithersburg, MD, USA). Each tissue core was 1.0 mm in diameter and 5.0 µm in thickness. The tumor node metastasis (TNM) classification, cancer grade, and androgen receptor (AR)/ER/PR/HER2 expression data related to the microarray were provided by the manufacturer.

### Ethics approval

The use of the microarray was approved by the Committee for the Ethics on the Experiments with Human Derivative Samples of National Institute of Advanced Industrial Science and Technology.

### rBC2LCN lectin histochemical staining

The rBC2LCN and BSA were labeled using a horseradish peroxidase labeling kit (LK11; Dojindo), as described previously [14]. Lectin histochemical staining was performed using human breast carcinoma tissue microarray. The carcinoma sections were dewaxed and hydrated in 10 mm citric acid (pH 6.0), followed by autoclaving at 120 °C for 10 min. The antigen‐activated sections were immersed in 0.3% hydrogen peroxide in methanol at room temperature for 10 min to block endogenous peroxidase activity. Thereafter, these sections were rinsed, microwaved in ethylenediaminetetraacetic acid buffer (1 mm, pH 8.0) for 10 min, equilibrated in PBS, blocked with 1% BSA at room temperature for 10 min, and incubated in 10 μg·mL^−1^ of HRP‐conjugated rBC2LCN at room temperature for 1 h. Subsequently, the sections were rinsed in PBS and distilled water and were stained with Histofine DAB substrate kit (425011; Nichirei Corporation, Tokyo, Japan). A negative control was processed in a similar way using HRP‐conjugated BSA.

Images were taken with a BIOREVO BZ‐9000 fluorescence microscope (Keyence, Osaka, Japan). The image obtained was converted to an 8‐bit type image with 256 gray levels, and then, the level of the histogram peak of the cancer region was taken as a signal value from 0 to 255 to quantify rBC2LCN signal intensity using imagej v1.5.0 software (Rasband, W.S.; NIH, Bethesda, MD, USA, http://imagej.nih.gov/ij/). rBC2LCN signal intensity was obtained by subtracting the value of the negative control from that of the rBC2LCN.

### Statistical analysis

One‐way analysis of variance (ANOVA), Fisher’s LSD, and Tukey’s HSD tests were performed using kaleidagraph v4.5.2 software (Synergy Software, Eden Prairie, MN, USA).

## Results

### rBC2LCN‐positive and rBC2LCN‐negative breast carcinoma cell lines

**Fig. 1:**
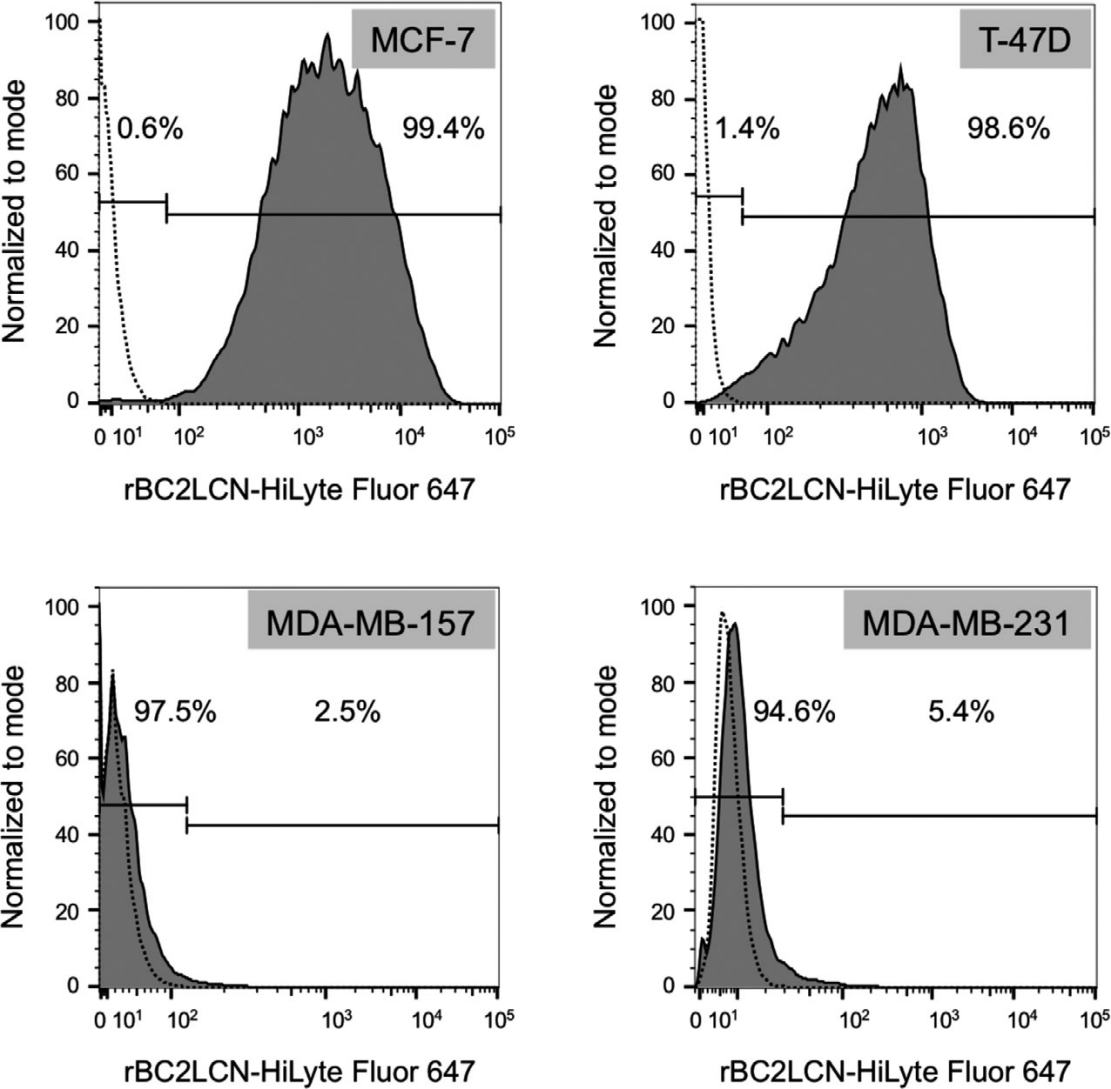
Flow cytometric analysis of human breast carcinoma cell lines using rBC2LCN. Histograms of MCF‐7, T‐47D, MDA‐MB‐157, and MDA‐MB‐231 cells incubated with 0.1 µg·mL^−1^ HiLyte Fluor 647‐conjugated rBC2LCN (solid line) or HiLyte Fluor 647‐conjugated BSA (dotted line, negative control). The ratios above and below the right side of the fluorescence intensity of the negative control were calculated as rBC2LCN‐positive and rBC2LCN‐negative proportions, respectively. Data are representative of three independent experiments.

To investigate the difference in the rBC2LCN lectin reactivity among the human breast carcinoma cell lines, we performed flow cytometric analyses of MCF‐7, T‐47D, MDA‐MB‐157, and MDA‐MB‐231 cells using rBC2LCN labeled with HiLyte Fluor 647 (Fig. 1). The rBC2LCN bound specifically to the MCF‐7 and T‐47D cells but not to the MDA‐MB‐157 and MDA‐MB‐231 cells. Based on the results, MCF‐7 and T‐47D were classified as rBC2LCN‐positive breast carcinoma cell lines, whereas MDA‐MB‐157 and MDA‐MB‐231 were classified as rBC2LCN‐negative cell lines.

### The rBC2LCN‐positive breast carcinoma cell lines showed higher expression levels of epithelial marker genes

**Fig. 2:**
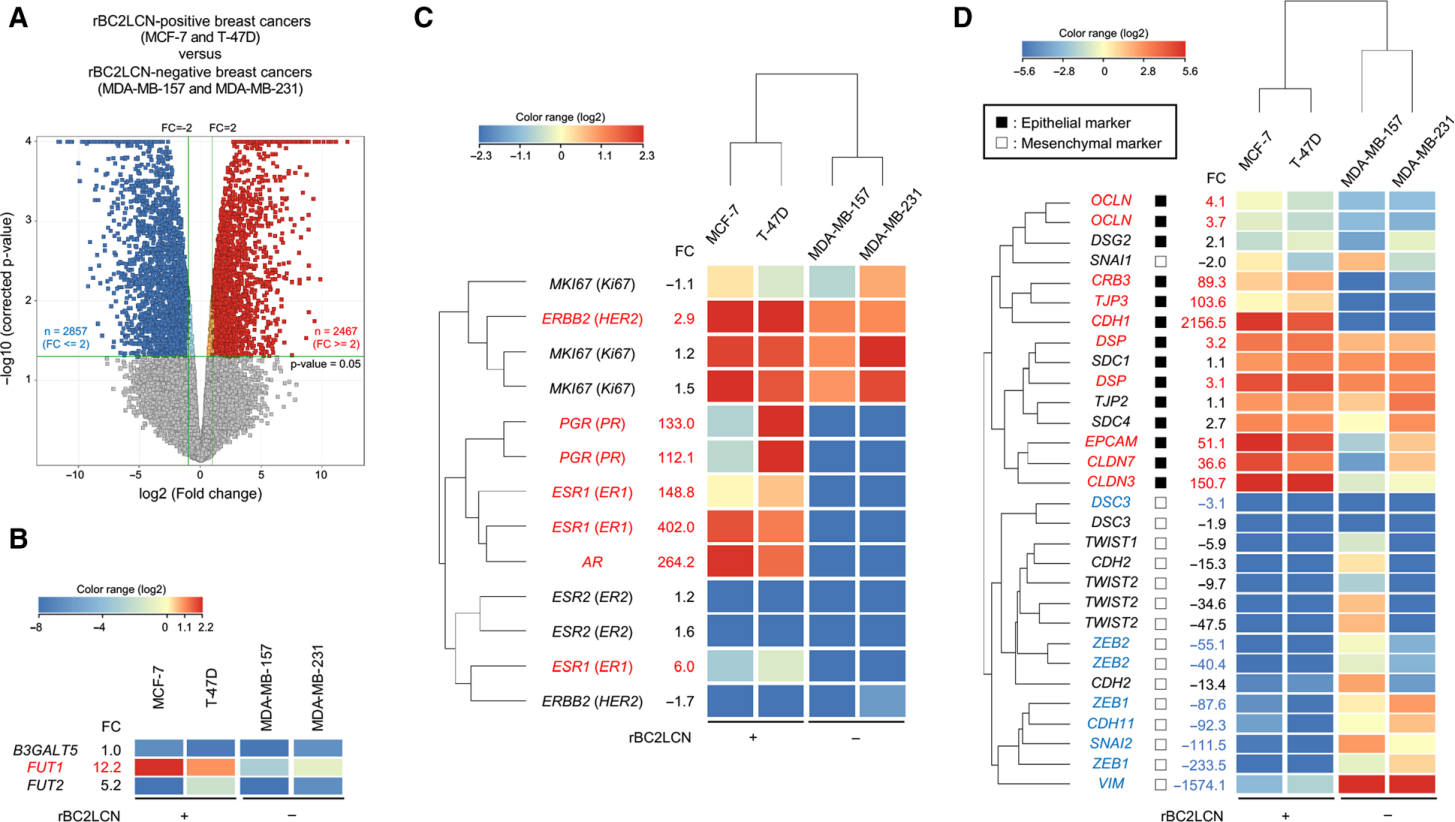
DNA microarray analysis of the rBC2LCN‐positive and rBC2LCN‐negative human breast carcinoma cell lines. (A) Volcano plot analysis of the rBC2LCN‐positive and rBC2LCN‐negative breast carcinoma cell lines. DNA microarray analysis is performed using rBC2LCN‐positive cell lines (MCF‐7, *n* = 2 and T‐47D, *n* = 2) and rBC2LCN‐negative cell lines (MDA‐MB‐157, *n* = 2 and MDA‐MB‐231, *n* = 2). The genes that show significant changes in the average expression levels between the rBC2LCN‐positive and the rBC2LCN‐negative cell lines are indicated by color plots (significance analysis: moderated *t*‐test; *P* value computation: asymptotic; multiple testing correction: Benjamini–Hochberg; corrected *P* value ≤ 0.05). The red and blue plots indicate 2.0‐fold higher and lower expression levels, respectively, in the rBC2LCN‐positive cell lines than in the rBC2LCN‐negative cell lines. Detailed data are shown in Tables S3 and S4. (B) Heat map showing rBC2LCN‐related glycosyltransferase genes. Fold changes (FC) between the rBC2LCN‐positive and the rBC2LCN‐negative cell lines are indicated. A red‐colored number indicates a significantly higher expression difference in the rBC2LCN‐positive cell lines than in the rBC2LCN‐negative cell lines. Detailed data are shown in Table S5. (C) Heat map showing the genes related to the breast cancer type. FC between the rBC2LCN‐positive and the rBC2LCN‐negative cell lines are indicated. Red‐colored numbers indicate significantly higher expression differences in the rBC2LCN‐positive cell lines than in the rBC2LCN‐negative cell lines. Clustering analysis is performed using a Euclidean distance measure and Ward’s linkage. Detailed data are shown in Table S6. (D) Heat map showing the epithelial and mesenchymal marker genes. FC between the rBC2LCN‐positive and the rBC2LCN‐negative cell lines are indicated. Red‐ and blue‐colored numbers indicate significantly higher and lower expression differences, respectively, in the rBC2LCN‐positive cell lines than in the rBC2LCN‐negative cell lines. Clustering analysis is performed using a Euclidean distance measure and Ward’s linkage. Detailed data are shown in Table S10.

We previously reported that the proportion of rBC2LCN‐positive cells of the human prostate carcinoma cell line PC‐3 decreased with increasing cell passages [12]. To compare the comprehensive expression profiles between the rBC2LCN‐positive and the rBC2LCN‐negative breast carcinoma cell lines, we performed DNA microarray analysis using the rBC2LCN‐positive (MCF‐7, *n* = 2 and T‐47D, *n* = 2) and the rBC2LCN‐negative (MDA‐MB‐157, *n* = 2 and MDA‐MB‐231, *n* = 2) cell lines under the same conditions illustrated in Fig. 1 (Table S2). Clustering analysis of all the genes spotted on the array clearly showed that the breast carcinoma cell lines were divided into rBC2LCN‐positive and rBC2LCN‐negative cell lines (Fig. S1). The volcano plot analysis detected 2467 and 2857 genes that were significantly more than two times upregulated and downregulated, respectively, in the rBC2LCN‐positive cell lines, compared with those in the rBC2LCN‐negative cell lines (Fig. 2A and Tables S3–S4). Glycosyltransferases, such as B3GalT5 and FUT1/2, were found to specifically synthesize glycans that were bound by rBC2LCN [9]. *FUT1* showed a significantly higher expression by 12.2 times in the rBC2LCN‐positive cell lines than that in rBC2LCN‐negative cells (Fig. 2B and Table S5). However, we did not observe any significant differences in the *B3GalT5* and *FUT2* gene expressions between the rBC2LCN‐positive and the rBC2LCN‐negative cell lines (Fig. 2B and Table S5). Significantly higher expressions of *AR*, *ER1*, *HER2*, and *PR* were observed in the rBC2LCN‐positive cell lines than in the rBC2LCN‐negative cell lines (Fig. 2C and Table S6).

Gene ontology enrichment analyses of 2467 upregulated and 2857 downregulated genes in the rBC2LCN‐positive cell lines were performed (Tables S7 and S8). Two epithelial‐to‐mesenchymal transition‐related GO terms (regulation of epithelial‐to‐mesenchymal transition, GO:0010717; positive regulation of epithelial‐to‐mesenchymal transition, GO:0010718) were significantly detected in the downregulated genes (Tables S8 and S9). The epithelial‐to‐mesenchymal transition was associated with increased aggressiveness and the invasive and metastatic potential in carcinomas [15]. Next, we focused on the typical epithelial and mesenchymal marker genes. The rBC2LCN‐positive cell lines showed high expressions of the epithelial marker genes *CDH1*, *CLDN3*, *CLDN7*, *CRB3*, *DSP*, *EPCAM*, *OCLN*, and *TJP3*, whereas the rBC2LCN‐negative cell lines showed high expressions of the mesenchymal marker genes *CDH11*, *DSC3*, *SNAI2*, *VIM*, *ZEB1*, and *ZEB2* (Fig. 2D and Table S10). In addition, five GO terms, which were related to the transforming growth factor β pathway, were detected in the biological processes of the downregulated genes (Tables S8 and S9). Notably, TGFβ induces the progression of cancer through the epithelial‐to‐mesenchymal transition [16]. These results indicated that rBC2LCN can distinguish between breast carcinoma cell lines that expressed epithelial markers and those that expressed mesenchymal markers.

### rBC2LCN lectin detected early‐stage HER2‐positive breast carcinoma

**Fig. 3:**
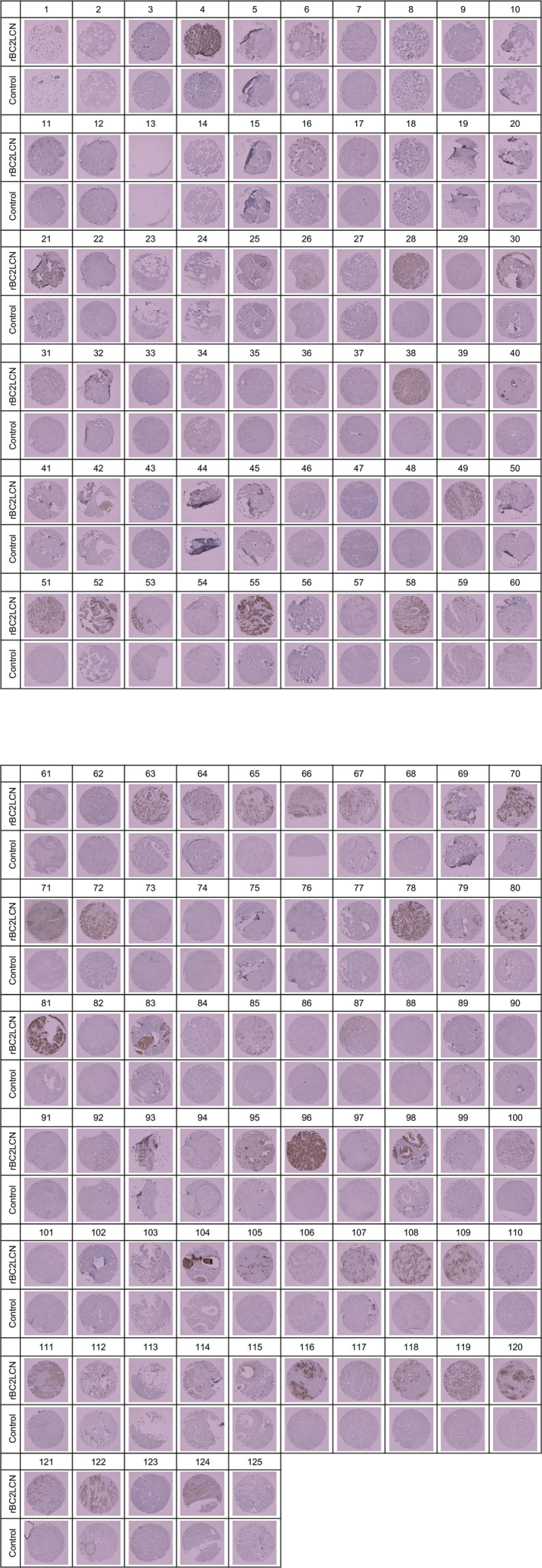
Histochemical staining with rBC2LCN lectin using a human breast carcinoma tissue microarray. The data on rBC2LCN signal intensity; TNM classification (T parameter: T1–T4; N parameter: N0–N3; M parameter: M0–M1); cancer grade (Grades I–III); and AR/PR/ER/HER2 expression levels that are related to the microarray are shown in Table S11.

To investigate the human breast cancer subtypes and stages that were detected by the rBC2LCN lectin, we performed histochemical staining using human breast carcinoma tissue microarray (Figs 3 and S2). Then, we quantified the rBC2LCN signal intensities on the human breast carcinoma tissue microarray using imagej software (Table S11). The TNM classification (T1–T4, N0–N3, and M0–M1), cancer grade (Grades I–III), and AR/PR/ER/HER2 expression level data that were related to the microarray were provided by the manufacturer (Table S11). The rBC2LCN signal intensities of the invasive ductal carcinoma tissue sections were classified by the data mentioned above (Fig. S3 and Table S11).

**Fig. 4:**
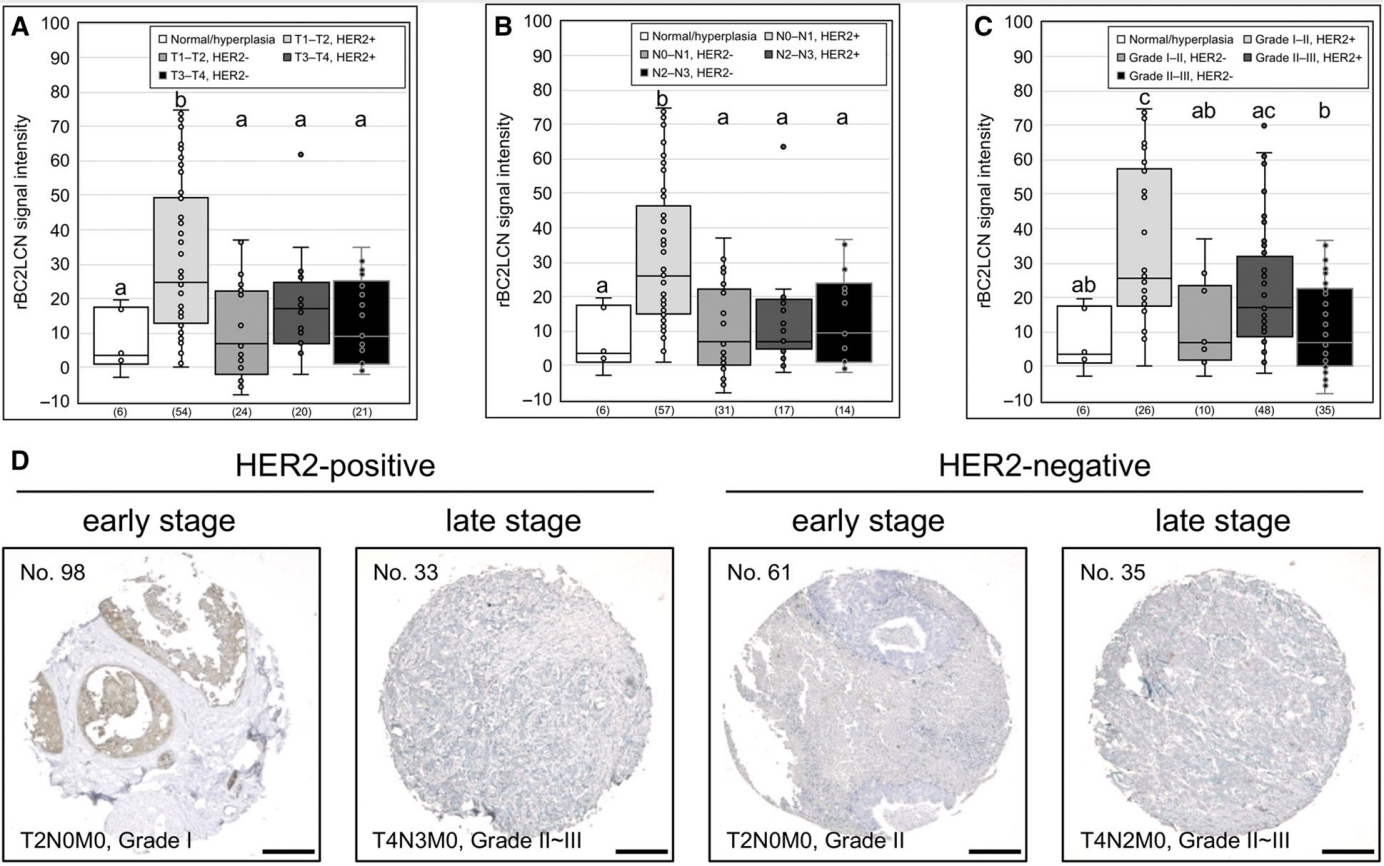
The rBC2LCN signal intensity and various parameters of human breast carcinoma. The rBC2LCN signal intensities of invasive ductal carcinoma tissues are quantified using imagej software and are classified by (A) T parameter and HER2 expression, (B) N parameter and HER2 expression, and (C) cancer grade and HER2 expression. T parameter, T1–4, represented the size or direct extent of the primary tumor (T1: smaller; T4: larger). The N parameter, N0–3, represented the degree of spread to regional lymph nodes (N0: no metastasis; N3: tumor spread to more distant or numerous regional lymph nodes). Cancer grade, Grades I–III, was assessed based on the cell appearance on pathology examination (I: well‐differentiated and slow‐growing; III: poorly differentiated and fast‐growing). Grades I–II represents Grade I and Grades I–II; Grades II–III represents Grade II, Grades II–III, and Grade III. One‐way ANOVA (*P* ≤ 0.05), followed by Tukey’s HSD test, was performed. Significant differences in the Tukey’s HSD test (*P* ≤ 0.05) are indicated by different letters (a, b, and c). The numbers analyzed are shown in parentheses. (D) Representative histochemical staining pictures of rBC2LCN. The scale bar indicates 200 μm.

The rBC2LCN signal intensities were significantly stronger in the tissues classified as T1–T2, N0–N1, Grades I–II, or HER2 + than in those classified as normal/hyperplasia and T3–T4, N2–N3, Grades II–III, or HER2− (Fig. S3A,B,D,H). However, there was no difference in the rBC2LCN signal intensities among the tissues that were classified by the M parameter and the AR, PR, and ER expressions (Fig. S3C,E,F,G). Next, the rBC2LCN signal intensities were classified according to the combinations of the T parameter and HER2 expression, N parameter and HER2 expression, or cancer grade and HER2 expression (Fig. 4). Interestingly, significantly higher intensities were seen in the categories of T1–T2/HER2+ and N0–N1/HER2+ (Fig. 4A,B). In addition, the rBC2LCN signal intensity was significantly higher for Grades I–II/HER2 + than for the others, except for one case of Grades II–III/HER2+ (Fig. 4C). Early‐stage cancer is defined as one that has not spread much in the early phase of growth. These results suggested that rBC2LCN lectin can specifically detect early‐stage HER2‐positive breast carcinoma (Fig. 4D).

## Discussion

The molecular characterization of the breast carcinoma cell lines, such as the luminal epithelial‐like and the mesenchymal/basal‐like cells, has led to the identification of novel markers for cancer diagnosis and targeted therapy [17]. Previously, we reported that as the cell passage increased, there was a decrease in the proportion of rBC2LCN‐positive cells in the human prostate carcinoma cell line PC‐3 with high expression levels of epithelial marker genes [12]. Generally, the gene expression profile is known to change according to the cell culture condition, as well as the cell culture passage. Therefore, in this study, flow cytometric and DNA microarray analyses were performed using breast carcinoma cells that were cultured under the same condition. The rBC2LCN‐positive cell lines, MCF‐7 and T‐47D, were found to highly express the epithelial marker genes, whereas the rBC2LCN‐negative cells, MDA‐MB‐157 and MDA‐MB‐231, were found to highly express the mesenchymal marker genes. Consistently, our results were in accordance with the transcriptomic results of the study by Charafe‐Jauffret *et al*. [17] on breast carcinoma cell lines. These findings indicated that rBC2LCN can aid in detecting carcinoma cells that have high expressions of epithelial marker genes.

Carbohydrate structures have been suggested to be associated with poor prognosis and reduced overall survival in breast cancer [18]. Globo H is one of the GalNAc‐containing glycans bound by the rBC2LCN; it was first identified in MCF‐7 cells and was found to play crucial roles in cancer stem cell properties and tumor progression in breast cancer [8, 9, 19, 20, 21]. Globo H expression is higher in MCF‐7 and T‐47D cells than in MDA‐MB‐231 [13, 19]. B3GALT5 and FUT1/FUT2 have been known to be involved in the biosynthesis of Globo H in breast carcinoma cell lines [19, 20]. In this study, we revealed that the rBC2LCN‐positive cell lines exhibited higher *FUT1* expression compared with that in the rBC2LCN‐negative cell lines, indicating that rBC2LCN can detect breast carcinoma cells with high *FUT1* expression. In fact, MDA‐MB‐157 and MDA‐MB‐231 cells are known to be more aggressive compared with MCF‐7 and T‐47D cells [22]; this implied that the rBC2LCN‐positive cells were not necessarily more aggressive compared with rBC2LCN‐negative cells. On the other hand, using MCF‐7, T‐47D, and MDA‐MB‐231 cells, Lai *et al*. [13] described that *FUT1* overexpression increased Globo H expression and the invasive/metastatic properties of cells *in vitro* and *in vivo*, whereas *FUT1* knockdown showed the opposite effects. Likewise, our previous study indicated that compared with the rBC2LCN‐negative subpopulation of PC‐3 cells, the rBC2LCN‐positive subpopulation of PC‐3 cells with high *FUT1* expression showed increased cell motility, anchorage‐independent growth, and drug resistance *in vitro* [12]. From these findings, we hypothesized that rBC2LCN has the potential to detect breast carcinoma cells that acquire aggressiveness secondary to high *FUT1* expression in breast carcinoma tissue. Overall, rBC2LCN reactivity may be correlated with the aggressiveness of some types of breast carcinoma cells, but this concept cannot be applied to all of the heterogeneous breast carcinoma cells.

The rBC2LCN histochemical staining of human breast carcinoma tissue microarray indicated that rBC2LCN could detect some HER2‐positive breast carcinoma tissues. Indeed, the expression levels of Globo H and HER2 in breast cancer tissues have been known to have no significant correlation [20]. Based on its ability to bind specifically to carcinoma cells with relatively high expressions of epithelial marker genes, rBC2LCN may be able to detect early‐stage cancer that is in the early phase of growth and has not spread extensively. For the above‐mentioned reasons, rBC2LCN lectin can specifically detect early‐stage HER2‐positive breast carcinoma tissues.

Lectins have been identified to be potentially useful for cancer diagnosis, therapy, and drug delivery [5, 6, 7]. The rBC2LCN‐toxin fusion protein has been shown to have significant antitumor effects both *in vitro* and *in vivo,* indicating the applicability of rBC2LCN as a drug delivery carrier [7]. Although the glycoproteins recognized by rBC2LCN in early‐stage HER2‐positive breast carcinoma cells remain unknown, these may be novel drug target candidates for breast carcinoma. Moreover, compared with antibodies, lectin is less costly because it is easy to produce in large amounts using *Escherichia coli* [23, 24]. Therefore, rBC2LCN may be used as a detection probe and a drug delivery carrier for early‐stage HER2‐positive breast carcinoma.

## Conflict of interest

The authors declare no conflict of interest.

## Author contributions

SM, YH, and YI designed the study. SM, HT, YO, and YA performed the biological experiments. SM analyzed the data. SM performed the bioinformatics analysis. SM, YH, and HT wrote the paper.

## Supporting information


**Fig. S1.** Clustering analysis of DNA microarray data of the rBC2LCN‐positive and ‐negative human breast carcinoma cell lines. DNA microarray analysis was performed using rBC2LCN‐positive cells (MCF‐7, *n* = 2 and T‐47D, *n* = 2) and rBC2LCN‐negative cells (MDA‐MB‐157, *n* = 2 and MDA‐MB‐231, *n* = 2). The gene expression data of each averaged value were used for the clustering analysis based on a Euclidean distance measure and Ward’s linkage.
**Fig. S2.** Representative images of histochemical staining with rBC2LCN lectin using human breast carcinoma. Histochemical staining with rBC2LCN lectin was performed using a human breast carcinoma tissue microarray. (A–C), normal/hyperplasia tissue. Hyperplasia of the breast tissue is a benign breast condition. (A, B) Most areas are not stained. (C) The ductal epithelial cell cytoplasm and cell membrane and the luminal surface are weakly stained. (D–H), invasive ductal carcinoma. Strong (D) and weak (E) signals are seen in the cytoplasm and cell membrane. (F, G) Most areas are not stained. (H) rBC2LCN‐positive and ‐negative cells are observed in the same section. (C′, D′, E′, and H′) show enlarged figures of (C, D, E, and H), respectively. The long and short scale bars indicate 200 and 20 μm, respectively.
**Fig. S3.** rBC2LCN signal intensity of human breast carcinoma. Histochemical staining with rBC2LCN lectin was performed using human breast carcinoma tissue microarray with TNM classification, cancer grade, and AR/ER/PR/HER2 expression data. The rBC2LCN signal intensities of invasive ductal carcinoma are classified by the (A) T parameter, (B) N parameter, (C) M parameter, (D) cancer grade, (E) AR expression, (F) PR expression, (G) ER expression, and (H) HER2 expression. The rBC2LCN signal intensity was quantified using imagej software. The T parameter, T1–4, represented the size or direct extent of the primary tumor (T1, smaller; T4, larger). The N parameter, N0–3, represented the degree of spread to regional lymph nodes (N0, no metastasis; N3, tumor spread to more distant or numerous regional lymph nodes). The M parameter, M0–1, represented the presence of distant metastasis (M0, no metastasis; M1, metastasis to distant organs). The cancer grade, Grades I–III, was assessed based on the cell appearance on pathology examination (I, well‐differentiated and slow‐growing; III, poorly differentiated and fast‐growing). Grades I–II represents Grade I and Grades I–II; Grades II–III represents Grade II, Grades II–III, and Grade III. One‐way ANOVA (*P* ≤ 0.05), followed by Fisher’s LSD test, was performed. Significant differences in the Fisher’s LSD test are indicated by asterisks. The numbers analyzed are shown in parentheses. ^*^ *P* ≤ 0.05, ^**^ *P* ≤ 0.01, ^***^ *P* ≤ 0.001

**Table S1.** Cell culture.
**Table S2.** List of DNA microarray data.
**Table S3.** Comprehensive expression analysis and comparison between rBC2LCN‐positive and rBC2LCN‐negative cell lines (*P* ≤ 0.05, FC ≥ 2).
**Table S4.** Comprehensive expression analysis and comparison between rBC2LCN‐positive and rBC2LCN‐negative cell lines (*P* ≤ 0.05, FC ≤ 2).
**Table S5.** Expression of rBC2LCN‐related genes.
**Table S6.** Expression of breast cancer type‐related genes.
**Table S7.** GO enrichment analysis of upregulated genes in rBC2LCN‐positive breast carcinoma cell lines.
**Table S8.** GO enrichment analysis of downregulated genes in rBC2LCN‐positive breast carcinoma cell lines.
**Table S9.** Cancer‐related GO terms detected in the GO enrichment analysis.
**Table S10.** Expressions of epithelial and mesenchymal marker genes.
**Table S11.** Information on human breast carcinoma tissue microarray.

## References

[1] Cho N (2016) Molecular subtypes and imaging phenotypes of breast cancer. Ultrasonography 35, 281–288. 27599892 10.14366/usg.16030 PMC5040136

[2] Shah D and Osipo C (2016) Cancer stem cells and HER2 positive breast cancer: the story so far. Genes Dis 3, 114–123. 30123819 10.1016/j.gendis.2016.02.002 PMC6095671

[3] Fragomeni SM , Sciallis A and Jeruss JS (2018) Molecular subtypes and local‐regional control of breast cancer. Surg Oncol Clin N Am 27, 95–120. 29132568 10.1016/j.soc.2017.08.005 PMC5715810

[4] Goldstein IJ , Hughes RC , Monsigny M , Osawa T and Sharon N (1980) What should be called a lectin? Nature 285, 66.

[5] Yau T , Dan X , Ng CC and Ng TB (2015) Lectins with potential for anti‐cancer therapy. Molecules 20, 3791–3810. 25730388 10.3390/molecules20033791 PMC6272365

[6] Coulibaly FS and Youan BBC (2017) Current status of lectin‐based cancer diagnosis and therapy. AIMS Mol Sci 4, 1–27.

[7] Shimomura O , Oda T , Tateno H , Ozawa Y , Kimura S , Sakashita S , Noguchi M , Hirabayashi J , Asashima M and Ohkohchi N (2018) A novel therapeutic strategy for pancreatic cancer: targeting cell surface glycan using rBC2LC‐N lectin‐drug conjugate (LDC). Mol Cancer Ther 17, 183–195. 28939555 10.1158/1535-7163.MCT-17-0232

[8] Sulák O , Cioci G , Delia M , Lahmann M , Varrot A , Imberty A and Wimmerová M (2010) A TNF‐like trimeric lectin domain from Burkholderia cenocepacia with specificity for fucosylated human histo‐blood group antigens. Structure 18, 59–72. 20152153 10.1016/j.str.2009.10.021

[9] Tateno H , Toyota M , Saito S , Onuma Y , Ito Y , Hiemori K , Fukumura M , Matsushima A , Nakanishi M , Ohnuma K *et al* (2011) Glycome diagnosis of human induced pluripotent stem cells using lectin microarray. J Biol Chem 286, 20345–20353. 21471226 10.1074/jbc.M111.231274 PMC3121447

[10] Onuma Y , Tateno H , Hirabayashi J , Ito Y and Asashima M (2013) rBC2LCN, a new probe for live cell imaging of human pluripotent stem cells. Biochem Biophys Res Commun 431, 524–529. 23321312 10.1016/j.bbrc.2013.01.025

[11] Breiman A , López Robles MD , de Carné Trécesson S , Echasserieau K , Bernardeau K , Drickamer K , Imberty A , Barillé‐Nion S , Altare F and Le Pendu J (2016) Carcinoma‐associated fucosylated antigens are markers of the epithelial state and can contribute to cell adhesion through CLEC17A (Prolectin). Oncotarget 7, 14064–14082. 26908442 10.18632/oncotarget.7476 PMC4924698

[12] Mawaribuchi S , Onuma Y , Aiki Y , Kuriyama Y , Mutoh M , Fujii G and Ito Y (2019) The rBC2LCN‐positive subpopulation of PC‐3 cells exhibits cancer stem‐like properties. Biochem Biophys Res Commun 515, 176–182. 31133376 10.1016/j.bbrc.2019.05.108

[13] Lai TY , Chen IJ , Lin RJ , Liao GS , Yeo HL , Ho CL , Wu JC , Chang NC , Lee AC and Yu AL (2019) Fucosyltransferase 1 and 2 play pivotal roles in breast cancer cells. Cell Death Discov 5, 74. 30854233 10.1038/s41420-019-0145-y PMC6403244

[14] Tateno H , Matsushima A , Hiemori K , Onuma Y , Ito Y , Hasehira K , Nishimura K , Ohtaka M , Takayasu S , Nakanishi M *et al* (2013) Podocalyxin is a glycoprotein ligand of the human pluripotent stem cell‐specific probe rBC2LCN. Stem Cells Transl Med 2, 265–273. 23526252 10.5966/sctm.2012-0154 PMC3659831

[15] Sarrió D , Rodriguez‐Pinilla SM , Hardisson D , Cano A , Moreno‐Bueno G and Palacios J (2008) Epithelial‐mesenchymal transition in breast cancer relates to the basal‐like phenotype. Cancer Res 68, 989–997. 18281472 10.1158/0008-5472.CAN-07-2017

[16] Miyazono K (2009) Transforming growth factor‐beta signaling in epithelial‐mesenchymal transition and progression of cancer. Proc Jpn Acad Ser B Phys Biol Sci 85, 314–323. 10.2183/pjab.85.314 PMC3621568 19838011

[17] Charafe‐Jauffret E , Ginestier C , Monville F , Finetti P , Adélaïde J , Cervera N , Fekairi S , Xerri L , Jacquemier J , Birnbaum D *et al* (2006) Gene expression profiling of breast cell lines identifies potential new basal markers. Oncogene 25, 2273–2284. 16288205 10.1038/sj.onc.1209254

[18] Cazet A , Julien S , Bobowski M , Burchell J and Delannoy P (2010) Tumour‐associated carbohydrate antigens in breast cancer. Breast Cancer Res 12, 204. 20550729 10.1186/bcr2577 PMC2917018

[19] Kannagi R , Levery SB , Ishigami F , Hakomori S , Shevinsky LH , Knowles BB and Solter D (1983) New globoseries glycosphingolipids in human teratocarcinoma reactive with the monoclonal antibody directed to a developmentally regulated antigen, stage‐specific embryonic antigen 3. J Biol Chem 258, 8934–8942. 6863318

[20] Chang WW , Lee CH , Lee P , Lin J , Hsu CW , Hung JT , Lin JJ , Yu JC , Shao LE , Yu J *et al* (2008) Expression of Globo H and SSEA3 in breast cancer stem cells and the involvement of fucosyl transferases 1 and 2 in Globo H synthesis. Proc Natl Acad Sci USA 105, 11667–11672. 18685093 10.1073/pnas.0804979105 PMC2575305

[21] Chuang PK , Hsiao M , Hsu TL , Chang CF , Wu CY , Chen BR , Huang HW , Liao KS , Chen CC , Chen CL *et al* (2019) Signaling pathway of globo‐series glycosphingolipids and β1,3‐galactosyltransferase V (β3GalT5) in breast cancer. Proc Natl Acad Sci USA 116, 3518–3523. 30808745 10.1073/pnas.1816946116 PMC6397564

[22] Dai X , Cheng H , Bai Z and Li J (2017) Breast cancer cell line classification and its relevance with breast tumor subtyping. J Cancer 8, 3131–3141. 29158785 10.7150/jca.18457 PMC5665029

[23] Tateno H , Onuma Y and Ito Y (2014) Live‐cell imaging of human pluripotent stem cells by a novel lectin probe rBC2LCN. Methods Mol Biol 1200, 313–318. 25117245 10.1007/978-1-4939-1292-6_26

[24] Hashim OH , Jayapalan JJ and Lee CS (2017) Lectins: an effective tool for screening of potential cancer biomarkers. PeerJ 5, e3784. 28894650 10.7717/peerj.3784 PMC5592079

