# Supplementary material for: rBC2LCN lectin as a potential probe of early‐stage HER2‐positive breast carcinoma
Source: FEBS Open Bio. 2020 May 5;10(6):1056–64. doi: 10.1002/2211-5463.12852 (PMC7262912; doi:10.1002/2211-5463.12852)
Supplement: Supplementary file 1 — Fig. S1. Clustering analysis of DNA microarray data of the rBC2LCN‐positive and ‐negative human breast carcinoma cell lines. DNA microarray analysis was performed using rBC2LCN‐positive cells (MCF‐7, n = 2 and T‐47D, n = 2) and rBC2LCN‐negative cells (MDA‐MB‐157, n = 2 and MDA‐MB‐231, n = 2). The gene expression data of each averaged value were used for the clustering analysis based on a Euclidean distance measure and Ward’s linkage. Fig. S2. Representative images of histochemical staining with rBC2LCN lectin using human breast carcinoma. Histochemical staining with rBC2LCN lectin was performed using a human breast carcinoma tissue microarray. (A–C), normal/hyperplasia tissue. Hyperplasia of the breast tissue is a benign breast condition. (A, B) Most areas are not stained. (C) The ductal epithelial cell cytoplasm and cell membrane and the luminal surface are weakly stained. (D–H), invasive ductal carcinoma. Strong (D) and weak (E) signals are seen in the cytoplasm and cell membrane. (F, G) Most areas are not stained. (H) rBC2LCN‐positive and ‐negative cells are observed in the same section. (C′, D′, E′, and H′) show enlarged figures of (C, D, E, and H), respectively. The long and short scale bars indicate 200 and 20 μm, respectively. Fig. S3. rBC2LCN signal intensity of human breast carcinoma. Histochemical staining with rBC2LCN lectin was performed using human breast carcinoma tissue microarray with TNM classification, cancer grade, and AR/ER/PR/HER2 expression data. The rBC2LCN signal intensities of invasive ductal carcinoma are classified by the (A) T parameter, (B) N parameter, (C) M parameter, (D) cancer grade, (E) AR expression, (F) PR expression, (G) ER expression, and (H) HER2 expression. The rBC2LCN signal intensity was quantified using imagej software. The T parameter, T1–4, represented the size or direct extent of the primary tumor (T1, smaller; T4, larger). The N parameter, N0–3, represented the degree of spread to regional lymph nodes (N0, no metastasis [file FEB4-10-1056-s001.docx]

**
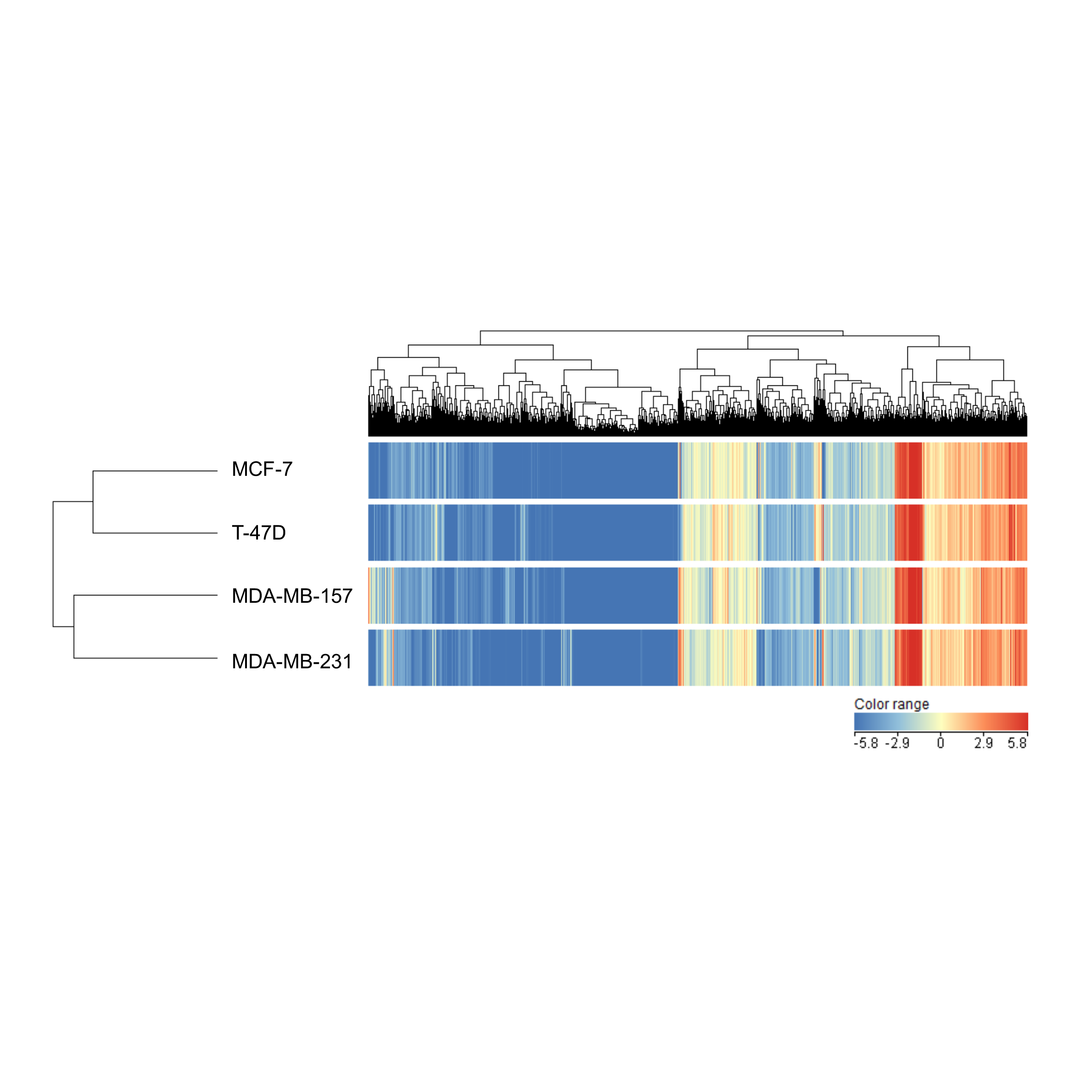
**

**Fig. S1. Clustering analysis of DNA microarray data of the rBC2LCN-positive and -negative human breast carcinoma cell lines**

DNA microarray analysis was performed using rBC2LCN-positive cells (MCF-7, n = 2 and T-47D, n = 2) and rBC2LCN-negative cells (MDA-MB-157, n = 2 and MDA-MB-231, n = 2). The gene expression data of each averaged value were used for the clustering analysis based on a Euclidean distance measure and Ward’s linkage.


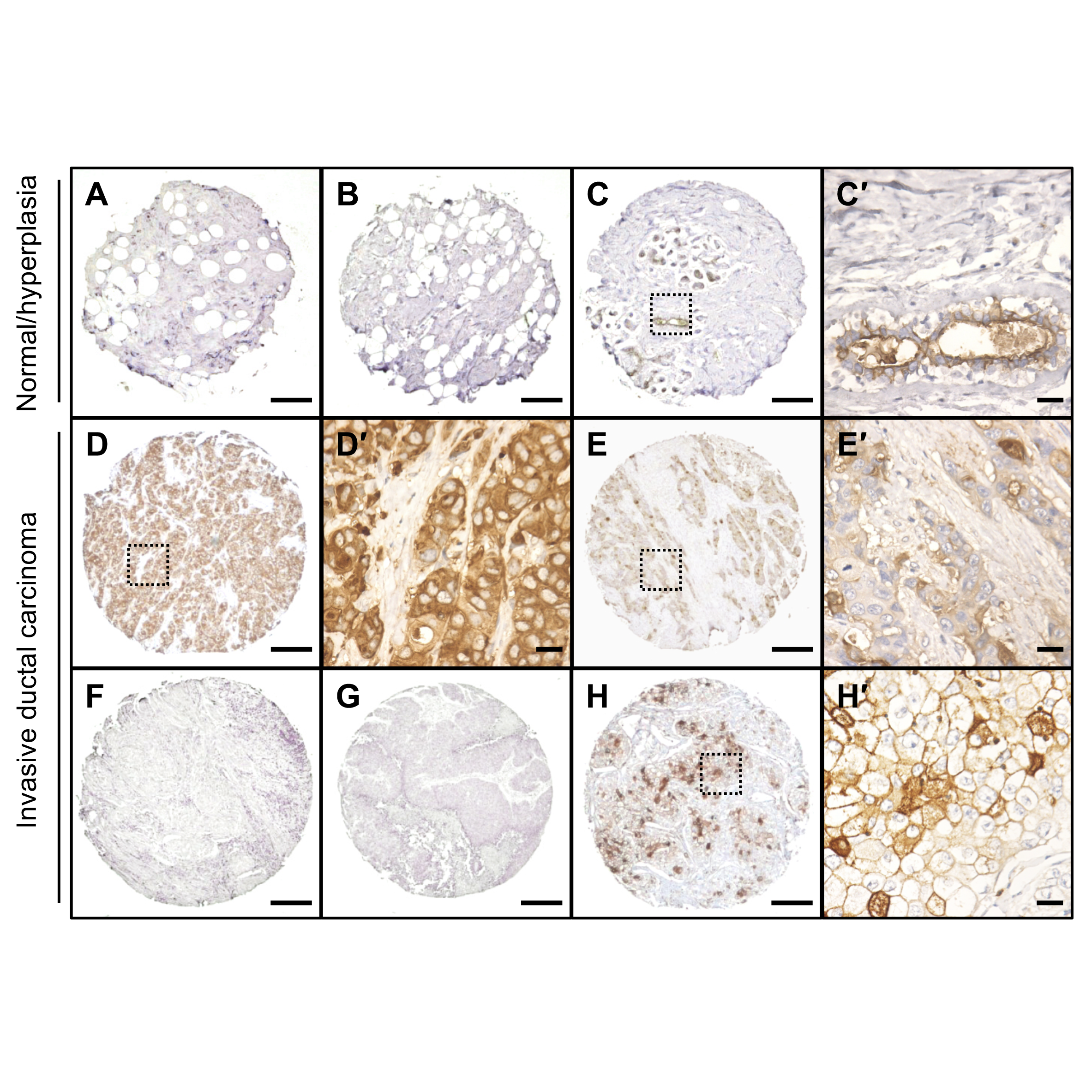


**Fig. S2. Representative images of histochemical staining with rBC2LCN lectin using human breast carcinoma**

Histochemical staining with rBC2LCN lectin was performed using a human breast carcinoma tissue microarray. (A–C), normal/hyperplasia tissue. Hyperplasia of the breast tissue is a benign breast condition. (A and B) Most areas are not stained. (C) The ductal epithelial cell cytoplasm and cell membrane and the luminal surface are weakly stained. (D–H), invasive ductal carcinoma. Strong (D) and weak (E) signals are seen in the cytoplasm and cell membrane. (F and G) Most areas are not stained. (H) rBC2LCN-positive and -negative cells are observed in the same section. (C′), (D′), (E′), and (H′) show enlarged figures of (C), (D), (E), and (H), respectively. The long and short scale bars indicate 200 μm and 20 μm, respectively.


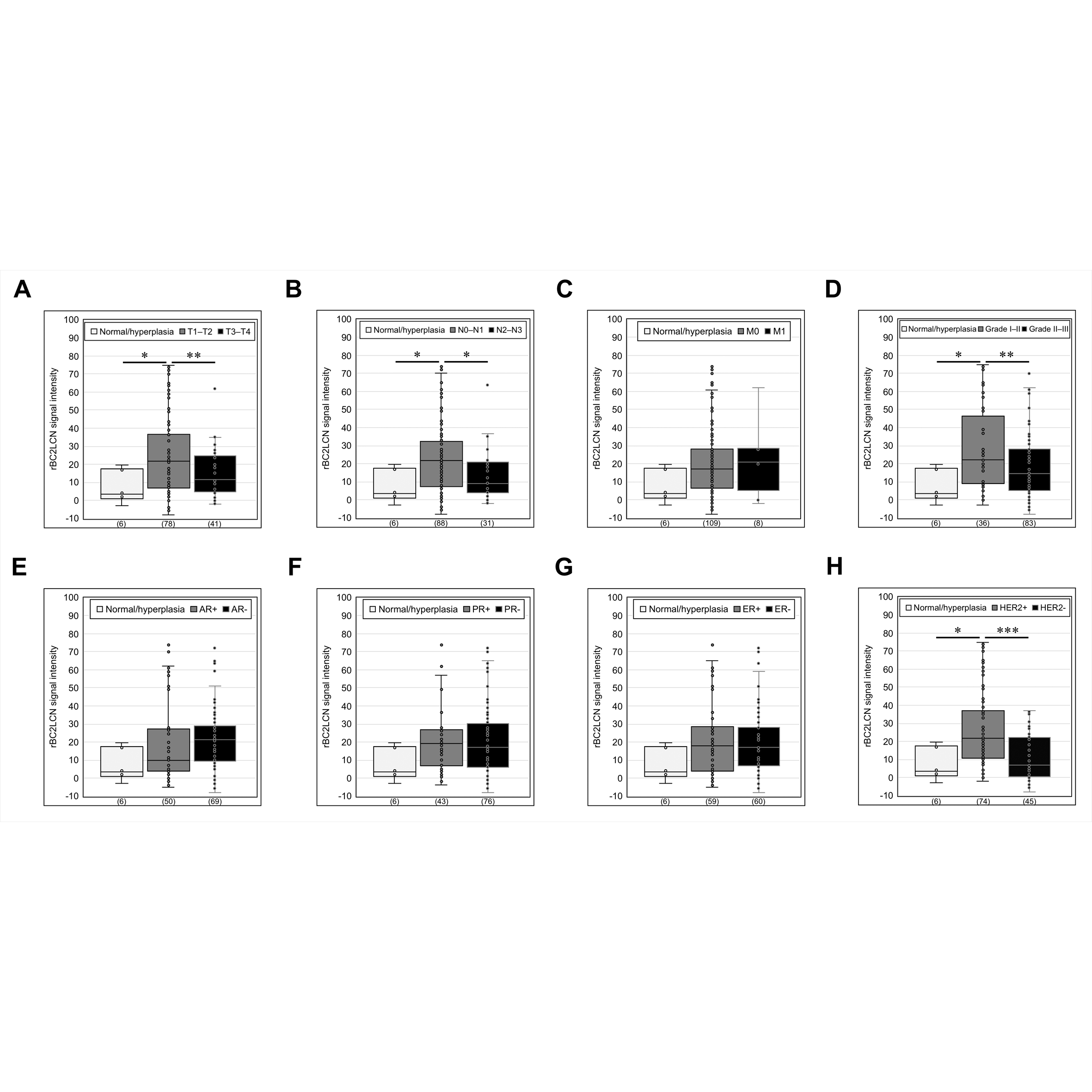


**Fig. S3. rBC2LCN signal intensity of human breast carcinoma**

Histochemical staining with rBC2LCN lectin was performed using human breast carcinoma tissue microarray with TNM classification, cancer grade, and AR/ER/PR/HER2 expression data. The rBC2LCN signal intensities of invasive ductal carcinoma are classified by the (A) T parameter, (B) N parameter, (C) M parameter, (D) cancer grade, (E) AR (androgen receptor) expression, (F) PR (progesterone receptor) expression, (G) ER (estrogen receptor) expression, and (H) HER2 (human epidermal growth factor receptor 2) expression. The rBC2LCN signal intensity was quantified using ImageJ software. The T parameter, T1–4, represented the size or direct extent of the primary tumor (T1, smaller; T4, larger). The N parameter, N0–3, represented the degree of spread to regional lymph nodes (N0, no metastasis; N3, tumor spread to more distant or numerous regional lymph nodes). The M parameter, M0–1, represented the presence of distant metastasis (M0, no metastasis; M1, metastasis to distant organs). The cancer grade, Grades I–III, was assessed based on the cell appearance on pathology examination (I, well-differentiated and slow-growing; III, poorly differentiated and fast-growing). Grades I–II represents Grade I and Grades I–II; Grades II–III represents Grade II, Grades II–III, and Grade III. One-way ANOVA (*P* ≤ 0.05), followed by Fisher’s LSD test, was performed. Significant differences in the Fisher’s LSD test are indicated by asterisks. The numbers analyzed are shown in parentheses.

^*^*P* ≤ 0.05, ^**^*P* ≤ 0.01, ^***^*P* ≤ 0.001
